# Comorbid Depressive and Anxiety Symptoms in a Patient with Myasthenia Gravis

**DOI:** 10.1155/2020/8967818

**Published:** 2020-01-08

**Authors:** Karthik R Cherukupally, Kodjovi Kodjo, Oluwaseun Ogunsakin, Olaniyi Olayinka, Patrice Fouron

**Affiliations:** Department of Psychiatry, Interfaith Medical Center, Brooklyn, New York, USA

## Abstract

**Introduction:**

Myasthenia gravis (MG) is a chronic illness most commonly found in women under 40 years. The most common psychiatric comorbidities found in MG include depressive and anxiety disorders.

**Clinical Presentation:**

We describe a case of a 43-year-old African American female with MG who was brought in for shortness of breath. History included MG diagnosed twelve years prior to the current presentation and a history of seven intubations. The patient was admitted to the ICU and intubated. She endorsed poor sleep, easy fatigability, and feeling hopeless in the context of psychosocial stressors—being single, homeless, and unemployed. The patient was started on Zoloft 50 mg per oral daily for depression and Atarax 50 mg per oral three times a day for anxiety. The patient responded well to the treatment and was discharged on day 10 after the resolution of her symptoms with appropriate aftercare in place.

**Discussion:**

Depressive and anxiety symptoms usually develop as comorbidity during MG disease. Depressive and anxiety symptoms, besides motor symptoms, have a negative impact on the quality of life. Mental health must be a clinical focus during the treatment of somatic symptoms during MG.

## 1. Introduction

Myasthenia gravis (MG) is a chronic autoimmune neuromuscular disease that causes weakness in the skeletal muscles resulting in difficulty in respiration and swallowing, diplopia, and ptosis. MG has a prevalence of 6/100000 [1]. The characteristic muscle weakness in MG is a weakness that worsens after periods of activity and improves after periods of rest. MG is caused by an error in the transmission of nerve impulses to muscles. It occurs when normal communication between the nerve and muscle is interrupted at the neuromuscular junction. Normally, when electrical signals or impulses travel down a motor nerve, the nerve endings release a neurotransmitter called acetylcholine. Acetylcholine travels from the nerve ending and binds to acetylcholine receptors on the muscle. The binding of acetylcholine to its receptor activates the muscle and causes muscle contraction. In MG, antibodies block, alter, or destroy the receptors for acetylcholine at the neuromuscular junction, which prevents the muscle from contracting. In most individuals with MG, this is caused by antibodies to the acetylcholine receptor itself. These antibodies are produced by the body's own immune system. All chronic diseases, including MG, potentially have psychiatric consequences in terms of coping and adaptation. Psychiatric morbidity usually appears as anxiety and depressive disorders such as panic disorder, generalized anxiety disorder, and depressive disorders. There are few data explaining the prevalence and association of many psychiatric symptoms among patients with MG. MG may not be the primary diagnosis initially because psychiatric symptoms may have similar presentations such as generalized muscle weakness, fatigue, and shortness of breath. Conversely, comorbid psychiatric symptoms that appear during the illness may be misdiagnosed as genuine myasthenic symptoms resulting in mistreatment. Consequently, there is a need for appropriate psychiatric treatment in order to avoid exacerbation of the underlying neurological symptoms [2]. MG patients sometimes present with symptoms of depression and anxiety. Furthermore, it is important to note that neurological disorders may present with symptoms of affection, cognition, and behavior. According to Craig, there is a 12% prevalence of consultation requests from neurology clinics and mood disorders are the most common comorbidities in neurological disorders [3, 4]. Symptoms of depression have been reported to be misdiagnosed and undertreated probably because the presentation of depression may overshadow mild symptoms of medical diseases or medical symptoms may overlap with the somatic symptoms of depression [5, 6]. Patients with more severe illnesses were reported to have higher levels of psychopathology than those with relatively less severe forms of the illness [7]. Findings on the relationship between the severity of MG and psychopathology seem inadequate and conflicting [8]. As a result of an increased spate of comorbid presentation, the interaction and association between MG and psychiatric disorders should be further evaluated. Our case report is aimed at evaluating and investigating the association between comorbid depression and anxiety symptoms among patients diagnosed with MG.

## 2. Clinical Presentation

We describe a case of a 43-year-old African American female with MG diagnosed twelve years prior to the current presentation and a history of seven intubations following acute crisis. The patient had a past medical history of seizure disorder, asthma, and diabetes mellitus. The patient was brought in for acute shortness of breath and was admitted to the intensive care unit for two days during which she was intubated. She was extubated on day 3 of admission and downgraded to the step down unit. On day 4, the patient had an exacerbation of her respiratory symptoms and was upgraded to the intensive care unit. She received pyridostigmine 60 mg per oral four times a day and prednisone 40 mg per oral daily that was subsequently tapered to 10 mg per oral daily. The consult-liaison team saw the patient on day 5 after the patient complained of feeling depressed and anxious. The patient endorsed poor sleep, easy fatigability, and feeling hopeless in the context of psychosocial stressors—being single, homeless, unemployed, and a burden to her family that worsened her anxiety. The patient reported anticipatory worry about her next myasthenia crisis that triggered her panic attacks which in turn worsened her shortness of breath. The patient reported that she used to benefit partially from anxiety and depressed mood by self-medicating with marijuana and cocaine. The patient reported a history of depression and anxiety occurring as a result of multiple hospitalizations and psychosocial stressors. She was on sertraline but has had been poorly compliant for the past 4 years. She reported intermittent suicidal ideations which worsened in the last six months. The patient was started on Zoloft 50 mg per oral daily for depression and Atarax 50 mg per oral three times a day for anxiety. The patient was not started on benzodiazepines for the risk of respiratory depression and to prevent future addiction to benzodiazepines as the patient has a history of substance use. The patient was downgraded to the floor on day 6. The patient responded well to treatment and was discharged on day 10 after the resolution of her symptoms with appropriate aftercare in place.

## 3. Discussion

Depressive and anxiety symptoms usually develop as comorbidity during MG disease [3]. The depressive state presents with mood changes, anhedonia, and feelings of helplessness and lack of confidence [9]. It is well established that depression is associated with immune dysregulation. There is strong evidence that depression involves alterations in multiple aspects of immunity that may contribute to the development or exacerbation of several medical disorders and may play a role in the pathophysiology of depressive symptoms [9]. Therefore, an inflammatory depression may require different therapeutic approaches than reactive depression in MG. Additionally, MG has a negative impact on the quality of life as they often experience unemployment, unwilling job transfers, and a decrease in income. In the case of our patient, the importance of psychosocial stressors should be underscored as they appeared to be one of the aggravating factors for the patient's symptoms. Many patients report feeling reduced social positivity. To inhibit the social disadvantages associated with MG and its treatment, greater focus needs to be placed on helping patients with MG resume a normal lifestyle as soon as possible by achieving the treatment target [10]. Mental health must be a clinical focus during the treatment of somatic symptoms during MG. Future research should be aimed at characterizing these subtypes better with the goal of optimizing treatment.
